# Hydroxyethyl starch-curcumin nanoparticles ameliorate DSS-induced ulcerative colitis in mice via synergistic TLR4/NF-κB suppression, Nrf2 activation, intestinal barrier restoration, and gut microbiota modulation

**DOI:** 10.3389/fphar.2025.1610711

**Published:** 2025-07-30

**Authors:** Sheng Li, Shiyi Lu, Jiahui Xiong, Liang Chen, Yanting Lin, Xiaohui Chen, Xinyi Wang, Lijing Lin, Xiaoliang Cai, Xiaoyu Yang

**Affiliations:** ^1^ Fuzhou Hospital of Traditional Chinese Medicine, Fuzhou, China; ^2^ Fujian University of Traditional Chinese Medicine, Fuzhou, China

**Keywords:** curcumin, ulcerative colitis, gut microbiota, TLR4/NF-κB, Nrf2

## Abstract

Here, we systematically evaluated the therapeutic efficacy and mechanisms of action of in-house synthesized hydroxyethyl starch-curcumin nanoparticles (HES-CUR NPs) in ulcerative colitis (UC). Using a dextran sodium sulfate (DSS)-induced UC mouse model, we analyzed the effect of HES-CUR NPs on colonic tissue and the gut microbiota through the assessment of organ indices and serum biochemical markers, histopathology, immunohistochemistry, Western blot, and 16S rRNA sequencing. We found that HES-CUR NPs significantly suppressed the TLR4/NF-κB inflammatory pathway (downregulated TLR4, p-IKKβ, IL-1β, IL-6, NFKB, MyD88, and p-IKKB/IKKB; p < 0.01), activated the Nrf2/HO-1 antioxidant pathway (enhanced Nrf2 nuclear translocation and HO-1 expression), and upregulated the expression of tight junction proteins (claudin-1, occludin, ZO-1), thereby restoring intestinal barrier integrity. Microbiota analysis revealed that HES-CUR NPs increased gut microbiota diversity (elevated Chao1, Shannon, and ACE indices) and enriched beneficial bacteria abundance (*Ligilactobacillus murinus*, *Lactobacillus johnsonii*). Antibiotic intervention partially attenuated the therapeutic effects of the HES-CUR NPs, confirming that their effects involved microbiota-dependent mechanisms. Compared to CUR and SASP, HES-CUR NPs exhibited significantly enhanced bioavailability and efficacy (p < 0.01), attributed to the targeted delivery and sustained-release properties of the hydroxyethyl starch nanocarrier. No toxicity was observed, as indicated by normal spleen, liver, and thymus indices and stable levels of TP, ALT, AST, ALB, GLB, and ALB/GLB. Our findings indicated that HES-CUR alleviates UC through a synergistic, multi-target mechanism involving inflammatory pathway suppression, antioxidant pathway activation, intestinal barrier repair, and microbiota modulation, providing a theoretical foundation for developing efficient and safe natural nanomedicines targeting UC.

## 1 Introduction

Ulcerative colitis (UC) is a subtype of chronic inflammatory bowel disease (IBD) characterized by recurrent inflammation of the colonic mucosal surface, with clinical manifestations including diarrhea, abdominal pain, and mucopurulent bloody stools (Wangchuk et al., 2024). UC has emerged as a global public health challenge owing to its high incidence, deleterious effect on patient quality of life, and strong link with colitis-associated colorectal carcinogenesis. However, the etiology of UC remains incompletely understood (Ng et al., 2017). Current research suggests that genetic predisposition, aberrant immune system activation, oxidative stress imbalance, and gut dysbiosis are key drivers of UC progression (Nan and Wang, 2023; Tan et al., 2023). Impaired intestinal mucosal barrier function is a critical pathological feature of this condition. The gut microecological balance plays a pivotal role in promoting intestinal epithelial cell proliferation, tight junction integrity, and the production of immunoglobulins and mucins, all of which are essential for the maturation and functional maintenance of the mucosal barrier (Quansah et al., 2023; Mohamad et al., 2021). The disruption of intestinal epithelial tight junction proteins increases intestinal permeability, facilitating the translocation of pathogens and their metabolites (e.g., lipopolysaccharide) into the submucosal layer, thereby activating innate immunity and triggering excessive inflammatory responses. Furthermore, the aberrant secretion of pro-inflammatory cytokines such as interleukin-1 beta (IL-1β), IL-6, and tumor necrosis factor-alpha (TNF-α) amplifies the inflammatory cascade. Although conventional therapeutics such as aminosalicylates and glucocorticoids provide temporary symptomatic relief, their long-term use often leads to drug resistance and systemic adverse effects, highlighting the urgent need for novel therapeutic strategies that combine efficacy with safety (Zeng et al., 2013; Tominaga et al., 2013; Thorlund et al., 2015; Toor et al., 2015). Current therapeutic strategies for IBD include biological agents (e.g., anti-TNF-α monoclonal antibodies) and functionalized bio-nanomaterials. While the former exhibits limitations such as low response rates and infection risks, the latter, despite their targeting potential, involve complex synthesis processes. In contrast, natural product-based nanoparticles offer superior advantages in safety and cost-effectiveness (Stojanov and Berlec, 2024; Tamilarasan et al., 2019).

Curcumin(CUR), a natural polyphenolic compound derived from the dried rhizomes of Curcuma longa and other species within the Zingiberaceae family (Murray-Stewart and Casero, 2017), exhibits notable anti-inflammatory (Ferguson et al., 2021), wound-healing (Salehi et al., 2021), antioxidant (Li H. et al., 2023), and antitumor (Murray-Stewart et al., 2018) properties, highlighting its potential as a therapeutic agent for ulcerative colitis (UC). However, its poor aqueous solubility, low bioavailability, and chemical instability severely restrict its clinical translation and applications in pharmaceutical development and functional foods (Tan et al., 2022). Nanodelivery systems have emerged as a groundbreaking strategy for addressing these limitations. The encapsulation of curcumin within lipid-based carriers, such as liposomes, polymeric nanoparticles, or micelles can significantly enhance its solubility, colon-targeting specificity, and mucosal-penetrating ability. For example, Zhu et al. (Zhu et al., 2023) constructed a quaternized chitosan-stabilized nanoemulsion system for curcumin delivery, which substantially improved the stability and bioavailability of this polyphenol. Similarly, Wang et al. (Wang et al., 2023) developed a curcumin-loaded polymeric mixed micelle system with anti-inflammatory activity, which demonstrated enhanced cumulative release *in vitro*, improved cellular uptake, and elevated oral bioavailability. In our previous study, curcumin was covalently conjugated with hydroxyethyl starch (HES) via esterification to synthesize pH-responsive nanoparticles (HES-CUR NPs), achieving a drug-loading efficiency (DLE) of 25.61%, far exceeding physical encapsulation methods. These nanoparticles enable targeted drug release in acidic tumor microenvironments, exerting dual-pathway antitumor effects through mitochondrial damage (ATP depletion, membrane potential collapse) and autophagy activation (upregulated Beclin-1 and LC3-II). Leveraging HES’s biocompatibility, they reduce toxicity while addressing limitations of conventional nanoparticles, such as low drug-loading capacity, poor stability, and single-mechanism therapeutic efficacy (Jiang et al., 2021). However, its therapeutic potential against UC remains unexplored.

Building on these advancements, in this study, we employed a dextran sulfate sodium (DSS)-induced murine colitis model to evaluate the anti-colitic therapeutic potential of the HES-CUR NP system. To achieve this, colonic tissue damage, serum inflammatory cytokine levels, and intestinal inflammatory factor expression were systematically analyzed. Furthermore, second-generation sequencing was used to characterize the changes in gut microbiota composition following HES-CUR NP administration and explore the interplay between inflammatory responses and microbiota alterations. The aim of this study was to elucidate the mechanisms by which the HES-CUR NPs alleviate UC symptoms, optimize the pharmacological activity of the system, and provide a theoretical foundation for developing curcumin-based functional products employing advanced nanodelivery systems.

## 2 Materials and methods

### 2.1 Materials

Kanamycin, tetracycline, and DSS were purchased from Shanghai Aladdin Biochemical Technology Co., Ltd.; curcumin was obtained from Shanghai Macklin Biochemical Technology Co., Ltd.; the H&E staining kit and the ELISA kits for IL-6(Product Code: E-MSEL-M0003), IL-1β(Product Code: E-MSEL-M0001), and TNF-α(Product Code: E-MSEL-M0002) were procured from Wuhan Procell Life Science and Technology Co., Ltd.

### 2.2 Preparation of the HES-CUR NPs

**FIGURE 1:**
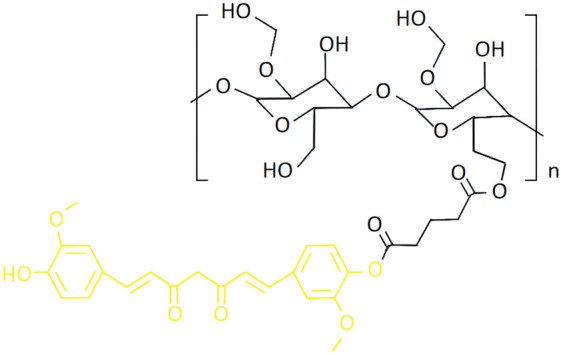
The molecular formula of HES-CUR NPs (Black: HES; Yellow: CUR) (Jiang et al., 2021).

The HES-CUR NP system (Figure 1) was prepared as detailed in our previous studies (Jiang et al., 2021; Xu et al., 2018; Chen et al., 2020).

#### 2.2.1 The synthesis of CUR-COOH

Step 1: Curcumin (4.42 g) was dissolved in pyridine (82 mL) and stirred for 15 min. Step 2) Glutaric anhydride (1.41 g) was dissolved in pyridine (10 mL). The resulting solution was gradually added to the solution from Step 1 under continuous stirring at room temperature for 7 h. After the reaction, the mixture was redissolved in ethyl acetate, washed three times with 1 mol/L HCl, and purified using silica gel column chromatography.

#### 2.2.2 The synthesis of HES-CUR

Step 1: HES-CUR polymer (5 mg) was dissolved in DMSO (1 mL). Step 2: HES (0.16 g) was dissolved in DMSO (2 mL). Then, 1-ethyl-3-(3-dimethylaminopropyl) carbodiimide (EDC) and 4-dimethylaminopyridine (DMAP) were added to the solution from Step 1 under stirring, followed by the dropwise addition of the solution from Step 2. The mixture was stirred at room temperature for 48 h, precipitated in a 1:1 (v/v) ether-ethanol mixture, and centrifuged. The precipitate was collected, washed three times, redissolved in DMSO, and dialyzed against ultrapure water for 3 days. The final product was lyophilized for 24 h, yielding HES-CUR as a yellow powder.

#### 2.2.3 Final preparation of HES-CUR NPs

HES-CUR polymer (5 mg) was dissolved in DMSO (1 mL). The solution was slowly injected into ultrapure water via a syringe pump under vigorous stirring. The mixture was dialyzed against ultrapure water for 24 h (water replaced every 6 h) and then lyophilized, yielding HES-CUR NPs as a yellow powder.

### 2.3 Experimental design and procedures

**FIGURE 2:**
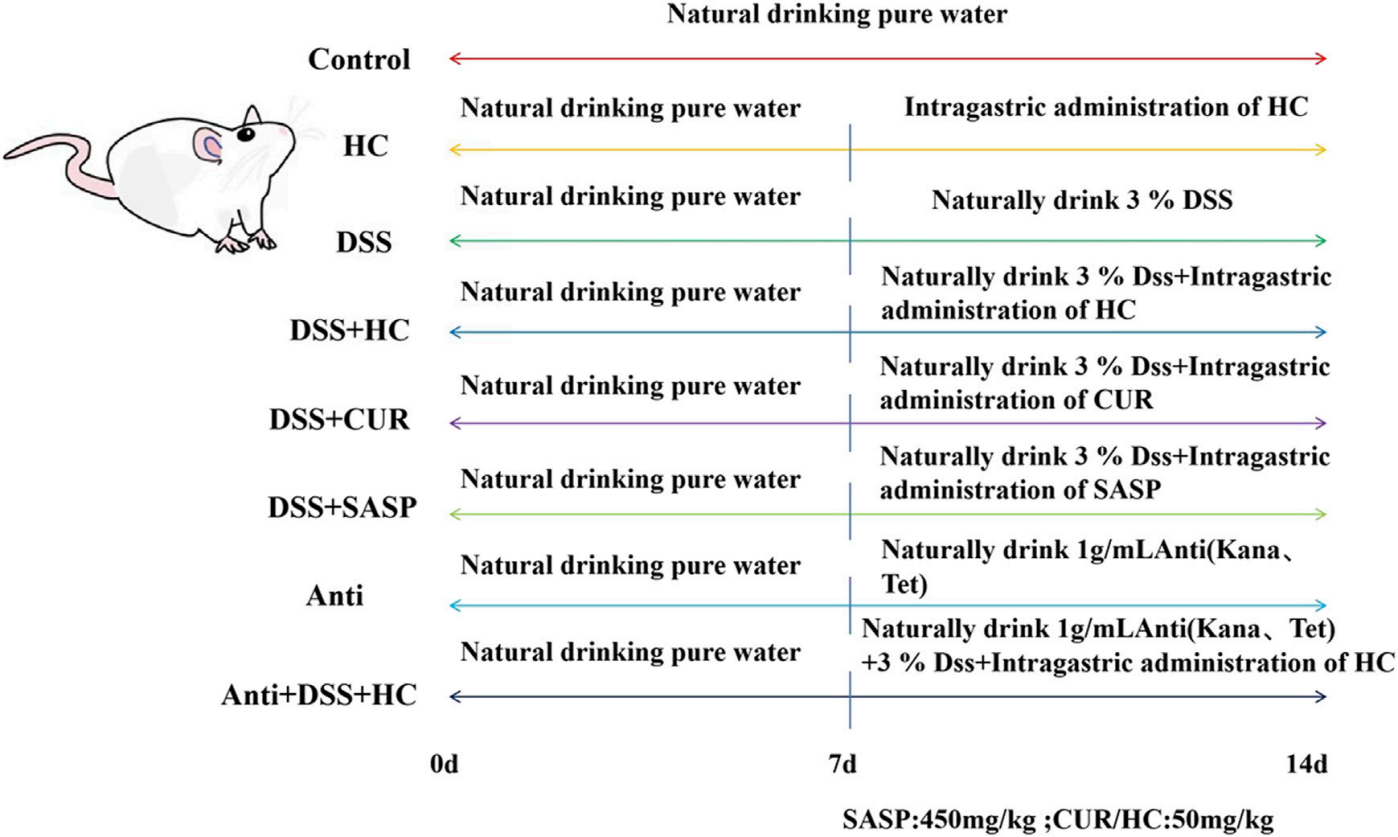
Animal experimental design.

A total of 80 specific pathogen-free (SPF) male Kunming mice (6 weeks old, weighing approximately 25 g) were provided by Fuzhou Nordons Biotechnology Co., Ltd. (Fuzhou, China). After 1 week of acclimatization under controlled conditions (temperature: 22°C ± 2°C; humidity: 50% ± 10%), mice were randomly divided into the following eight groups (n = 10 per group) based on the method described by Xu et al. (Xu et al., 2023), with minor modifications: Control, HES-CUR NPs(HC), DSS, DSS + HES-CUR NPs, DSS + CUR, DSS + Salazosulfapyridine (SASP, positive control drug), Antibiotic(Anti), and Antibiotic + DSS + HES-CUR NPs. The dosing regimen followed the schematic in Figure 2 and body weight was recorded daily.

### 2.4 Sample collection and processing

At the endpoint, blood was collected via retro-orbital bleeding and centrifuged at 9,500 rpm for 5 min. The obtained serum was stored at −80°C for biochemical analysis. Mice were euthanized by cervical dislocation, after which fecal samples (0.18–0.22 g) and tissues (liver, ileum, thymus, spleen) were rapidly collected. The colon was measured from the ileocecal junction to the anus, cut into 1-cm segments, and either fixed in 4% paraformaldehyde or frozen at −80°C. The organs were rinsed with saline until blood-free, blotted dry, and weighed. Organ indices were calculated as organ weight (mg)/body weight (g) and presented as mg/g.

### 2.5 Histopathological evaluation by H&E staining

To evaluate colon histopathology, tissues fixed in 4% paraformaldehyde were paraffin-embedded, sectioned, stained with H&E, and assessed for crypt architecture disruption, inflammatory cell infiltration, and epithelial damage across experimental groups under a light microscope.

### 2.6 Immunohistochemical (IHC) staining

After fixation in 4% neutral buffered formalin, tissues were deparaffinized, rehydrated, subjected to antigen retrieval, and blocked with serum. Based on preliminary optimization, the sections were incubated first with rabbit anti-ZO-1 (1:400 dilution) and rabbit anti-occludin (1:400 dilution) polyclonal antibodies overnight at 4°C, and then with an IHC secondary antibody (SP-9001). DAB chromogen (ZLI-9017) was employed for color development.

### 2.7 Western blot

Colonic tissue samples were thoroughly homogenized, and total protein was extracted using a Total Protein Extraction Kit (Cowin Century Co., Ltd., Beijing, China). Protein concentrations were determined using a BCA Protein Assay Kit (Shanghai Biyuntian Biotechnology Co., Ltd., Shanghai, China). Subsequently, 30 µg of protein per sample was separated by SDS-PAGE (Cowin Century Co., Ltd.), transferred onto PVDF membranes, blocked for 1 h, and incubated with primary antibodies overnight at 4°C. The following day, the membranes were washed with TBST buffer to remove unbound primary antibodies, and then incubated with the corresponding secondary antibodies for 1 h at room temperature. After washing off excess secondary antibodies, the protein bands were visualized using Enhanced Chemiluminescence (ECL) Reagent (Thermo Fisher Scientific, United States). Finally, the chemiluminescent signals were imaged using a gel imaging system, and the expression levels of the target proteins were determined.

### 2.8 Measurement of serum inflammatory cytokine levels in mice

The serum levels of IL-1β(MS Mouse IL-1β(Interleukin 1 Beta) ELISA Kit), IL-6(MS Mouse IL-6(Interleukin 6) ELISA Kit), and TNF-α (MS Mouse TNF-α(Tumor Necrosis Factor Alpha) ELISA Kit) were quantified using the respective ELISA kits according to the manufacturer’s instructions.

### 2.9 Determination of gut microbiota in mouse feces

For assessment of the fecal microbiota, total bacterial DNA was extracted from mouse feces according to the instructions provided in the fecal genomic DNA extraction kit(Biomarker Soil Genomic DNA Kit, Product Code: RK02005). Before PCR amplification, the purity and concentration of the DNA were determined by agarose gel electrophoresis and NanoDrop spectrophotometer, respectively.

### 2.10 Statistical analysis

Data were analyzed in SPSS20 and are presented as the means ± standard deviation of three or more parallel experiments. Origin2020 software was used for plotting. Statistical significance was assessed by one-way analysis of variance (ANOVA) or the t-test, as appropriate. P-values <0.05 were considered significant.

## 3 Result

### 3.1 HES-CUR NPs did not affect the immune organs and livers of mice

**FIGURE 3:**
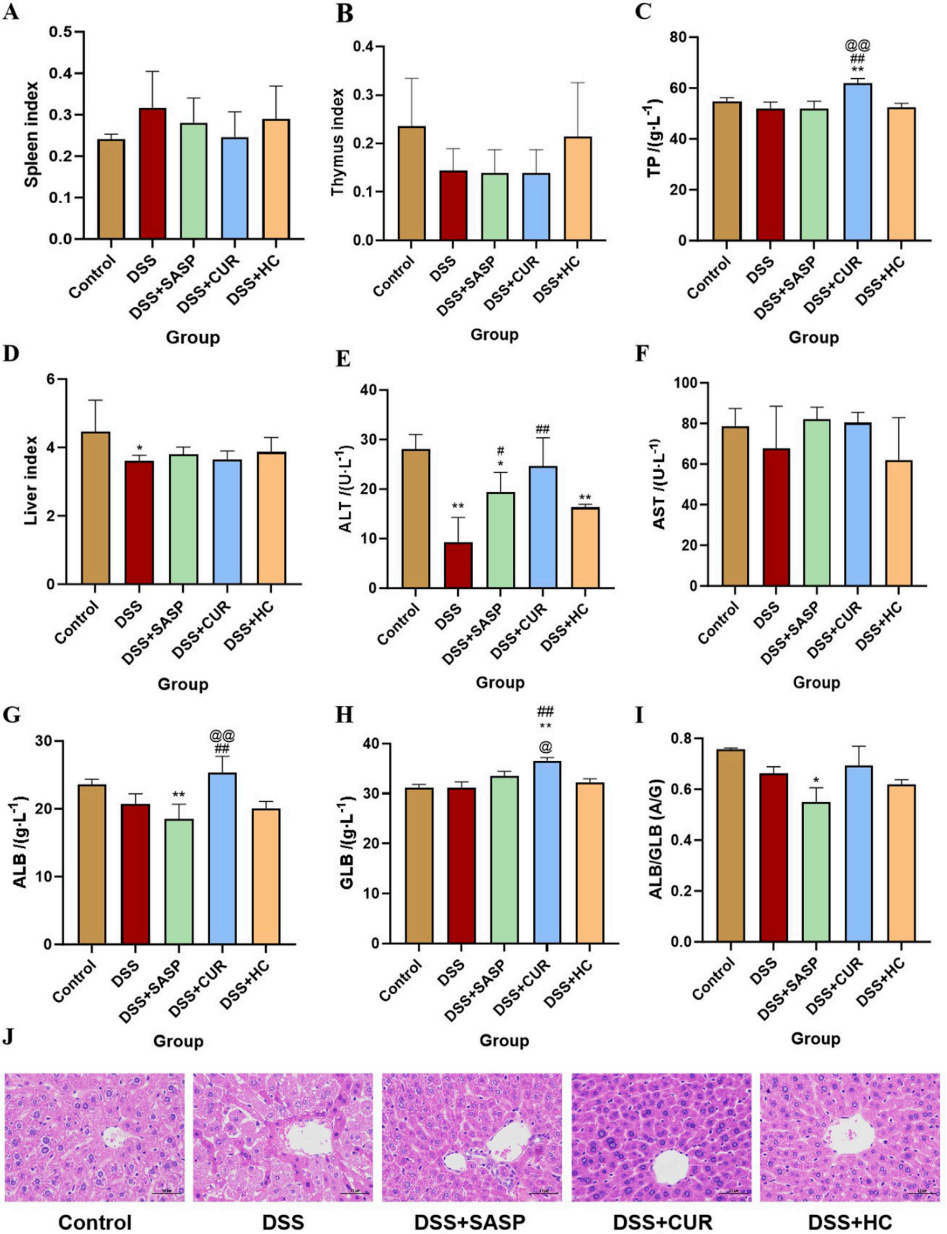
The effects of HES-CUR NPs on immune organs and the liver in ulcerative colitis (UC) model mice. **(A)** The effect of HES-CUR NPs on the spleen index, **(B)** the thymus index, **(C)** total protein (TP), **(D)** liver index, **(E)** alanine aminotransferase (ALT) levels, **(F)** aspartate aminotransferase (AST) levels, **(G)** albumin (ALB) levels, **(H)** globulin (GLB) levels, and **(I)** the ALB/GLB ratio. **(J)** Representative hematoxylin and eosin (H&E)-stained histological sections of mouse liver. *p < 0.05, **p < 0.01, compared with the Control group; #p < 0.05, ##p < 0.01, compared with the DSS group; @p < 0.05, @@p < 0.01, compared with the SASP group. Similar annotations apply to subsequent figures.

As shown in Figures 3A–I, compared with the Control group, the liver index and alanine aminotransferase (ALT) levels in the DSS group significantly decreased (p < 0.05), whereas HES-CUR NP treatment partially reversed this decline. No statistically significant difference in the splenic, hepatic, or thymic index was observed between the HES-CUR NP group and the Control group (p > 0.05), indicating that the HES-CUR NPs did not induce pathological hypertrophy or atrophy in immune organs (spleen, thymus) or the liver. The levels of liver function markers, including ALT and aspartate aminotransferase (AST), in the HES-CUR NP group remained comparable to those of the Control group, suggesting that the NPs did not induce hepatocyte injury or an inflammatory response. Plasma total protein levels, albumin levels, and the albumin-to-globulin (A/G) ratio remained stable, further confirming that synthetic function in the liver was unaltered and that protein metabolism was not impaired. Consistent with the biochemical findings, histopathological analysis (Figure 3J; Table 1) revealed intact hepatic lobule architecture and well-organized hepatocyte alignment in hematoxylin and eosin (H&E)-stained liver sections, with no evidence of steatosis, necrosis, or inflammatory infiltration.

**TABLE 1:** Histopathological Scoring of Mouse Liver Tissue (Mean ± SEM, n = 9).

Group	Score
Control	1
Model (DSS)	2.89±0.20**
DSS-CUR	1.56±0.18*^##^
DSS-HC	1.22±0.15^##^
DSS-SASP	1.33±0.17^##^

*p < 0.05, **p < 0.01, compared with the Control group; ^#^p < 0.05, ^##^p < 0.01, compared with the Model; ^@^p < 0.05, ^@@^p < 0.01, compared with the DSS-SASP group.

Grade I: Normal hepatic lobule structure, orderly arranged hepatic cords, without significant degeneration, necrosis or inflammatory cell infiltration. Score: 1 point; Grade II: Basically normal hepatic lobule structure, orderly arranged hepatic cords, occasional hepatocyte swelling, ballooning degeneration or steatosis, accompanied by mild inflammatory cell infiltration. Score: 2 points; Grade III: Disordered arrangement of hepatic cords, partial hepatocyte swelling, ballooning degeneration or steatosis, accompanied by inflammatory cell infiltration. Score: 3 points; Grade IV: Disordered arrangement of hepatic cords, hepatocyte swelling, ballooning degeneration or steatosis, accompanied by extensive inflammatory cell infiltration. Score: 4 points (Hyunna et al., 2022).

### 3.2 HES-CUR NPs alleviated UC symptoms in mice

**FIGURE 4:**
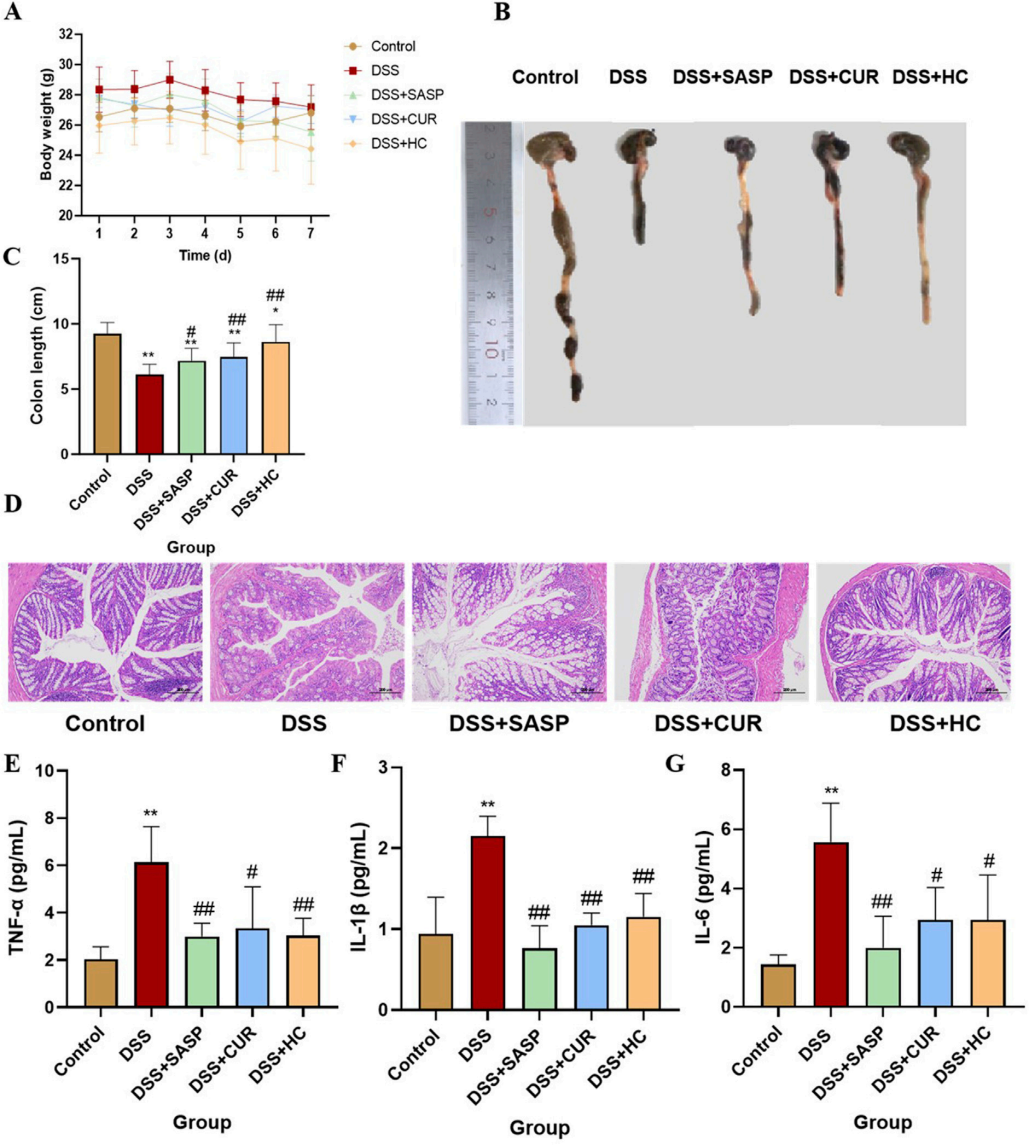
The therapeutic effects of HES-CUR NPs on ulcerative colitis (UC) model mice. **(A)** Body weight changes in mice. **(B,C)** Colon length alterations. **(D)** Representative hematoxylin and eosin (H&E)-stained histological sections of colonic tissues (×200 magnification). **(E)** TNF-α mRNA expression. **(F)** IL-1β mRNA expression. **(G)** IL-6 mRNA expression.

As shown in Figure 4A, no significant difference in weight was observed between the groups of mice. Colonic Macroscopic Damage Index (CMDI) scores (e.g., Table 2) confirmed the absence of colon injury in the Control group. In comparison, mice in the DSS group displayed pronounced congestion, edema, necrosis, and ulcer formation (maximum longitudinal diameter of approximately 1 cm) on the mucosal surface, along with intestinal wall thickening and inflammation. Notably, SASP, CUR, and HES-CUR NP treatments significantly ameliorated these pathological alterations (p < 0.01), with HES-CUR NPs achieving the most pronounced therapeutic effect, restoring colonic integrity to levels comparable (p > 0.05) to those of the Control group. The morphology of the colon was evaluated by H&E staining (Figure 4D). In the Control group, the epithelial structure of the colon was intact, cells were neatly arranged, and no inflammatory cell infiltration or ulceration was detected. In contrast, in the DSS model group, the colonic mucosal epithelium showed detachment and a disorganized cell arrangement, accompanied by mucosal and submucosal congestion, edema, massive infiltration of inflammatory cells, and diffuse distribution of small ulcers. Importantly, the DSS-induced colon tissue damage was significantly alleviated in the SASP, CUR, and HES-CUR NP treatment groups, with the most notable effect being achieved in the latter group. Additionally, the levels of IL-6, IL-1β, and TNF-α were significantly higher in the DSS model group than in the Control group (p < 0.05), with increases of 4.09 pg/mL, 1.21 pg/mL, and 4.09 pg/mL, respectively (Figures 4E–G), consistent with the CMDI and H&E staining results. This demonstrated that modeling in the mice had been successful. Compared with the DSS model group, the levels of these three cytokines were reduced in colon tissues from the SASP, CUR, and HES-CUR NP groups. In the SASP group, TNF-α, IL-1β, and IL-6 decreased by 3.13 pg/mL, 1.39 pg/mL, and 3.56 pg/mL, respectively, compared to the model group; In the CUR group, these cytokines were reduced by 2.79 pg/mL (TNF-α), 1.10 pg/mL (IL-1β), and 2.63 pg/mL (IL-6); In the HES-CUR NP group, the reductions reached 3.10 pg/mL (TNF-α), 1.00 pg/mL (IL-1β), and 2.63 pg/mL (IL-6). As depicted in Figures 4B,C, the colon length in mice of the DSS group was significantly shorter (p < 0.01) than that in mice of the Control, DSS + CUR, and DSS + HES-CUR NP groups. Moreover, the therapeutic effect was more pronounced in the DSS + HES-CUR NP group than in the DSS + CUR group, implying that our NP system relieved UC in mice, and to a greater than either SASP or free curcumin.

**TABLE 2:** Colonic Macroscopic Damage Index (CDMI) for mice in the different groups (n = 10, x ± 10).

Group	CMDI score
Control	0
Model (DSS)	3.90±0.88**
DSS-CUR	2.60±1.84**^@@^
DSS-HC	0.80±0.79^##@@^
DSS-SASP	2.20±1.32**^##^

*p < 0.05, **p < 0.01, compared with the Control group; ^#^p < 0.05, ^##^p < 0.01, compared with the Model; ^@^p < 0.05, ^@@^p < 0.01, compared with the DSS-SASP group.

Colonic Macroscopic Damage Index is a macroscopic assessment index for gross specimens that employs the following scoring criteria: 0: no visible damage; 1: mild hyperemia and edema, smooth mucosal surface, absence of erosions or ulcers; 2: moderate hyperemia and edema, mucosal roughness with granular appearance, presence of erosions or intestinal adhesions; 3: severe hyperemia and edema, mucosal necrosis with ulceration (maximum longitudinal ulcer diameter <1 cm), bowel wall thickening, or localized necroinflammation.; 4: the features of score 3 plus ulcers with maximum longitudinal diameters ≥1 cm or transmural necrosis.

### 3.3 HES-CUR NPs improved intestinal barrier function in UC model mice

**FIGURE 5:**
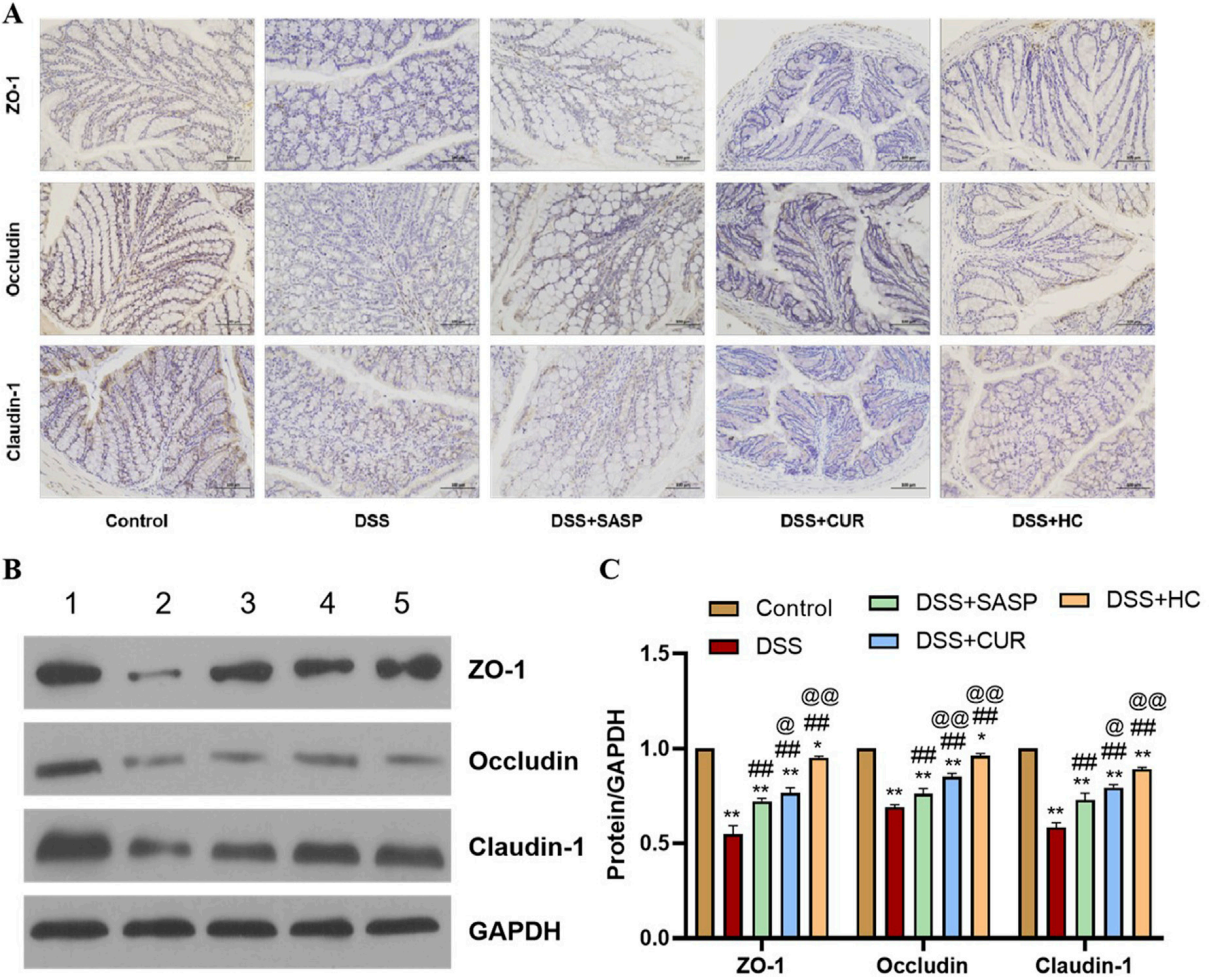
The effects of HES-CUR NPs on intestinal barrier function-related proteins in the colon of DSS-treated mice. **(A)** Representative immunohistochemical staining of mucosal barrier proteins in colonic tissues. **(B)** Western blot analysis of ZO-1, occludin, and claudin-1 protein expression in colonic tissues (1: Control; 2: DSS; 3: DSS+SASP; 4: DSS+CUR; 5: DSS+HC). **(C)** Quantitative comparison of ZO-1, occludin, and claudin-1 positivity across experimental groups.

As shown in Figure 5, the expression levels of ZO-1, occludin, and claudin-1 were significantly decreased in the DSS group compared with those of the Control group (p < 0.01). In contrast, SASP, CUR (free curcumin), and HES-CUR NP treatments markedly upregulated the expression of these tight junction proteins relative to that in the DSS group (p < 0.01), demonstrating their efficacy in repairing DSS-induced intestinal barrier damage and restoring tight junction functionality. Notably, the upregulation of the levels of ZO-1, occludin, and claudin-1 was more marked in the CUR and HES-CUR NP groups than in the SASP group (p < 0.05), indicative of the superior pharmacological activity of curcumin in mitigating intestinal barrier injury. Among the treatments, HES-CUR NPs showed the most pronounced intestinal barrier restorative effects, which may be attributed to the enhanced curcumin bioavailability and targeting capability conferred by the nanoformulation. These results collectively highlighted the significant capability of HES-CUR NPs to restore tight junction protein expression and reinforce intestinal barrier integrity.

### 3.4 HES-CUR NPs alleviated UC by suppressing TLR4/NF-κb and activating the Nrf2 antioxidant pathway

**FIGURE 6:**
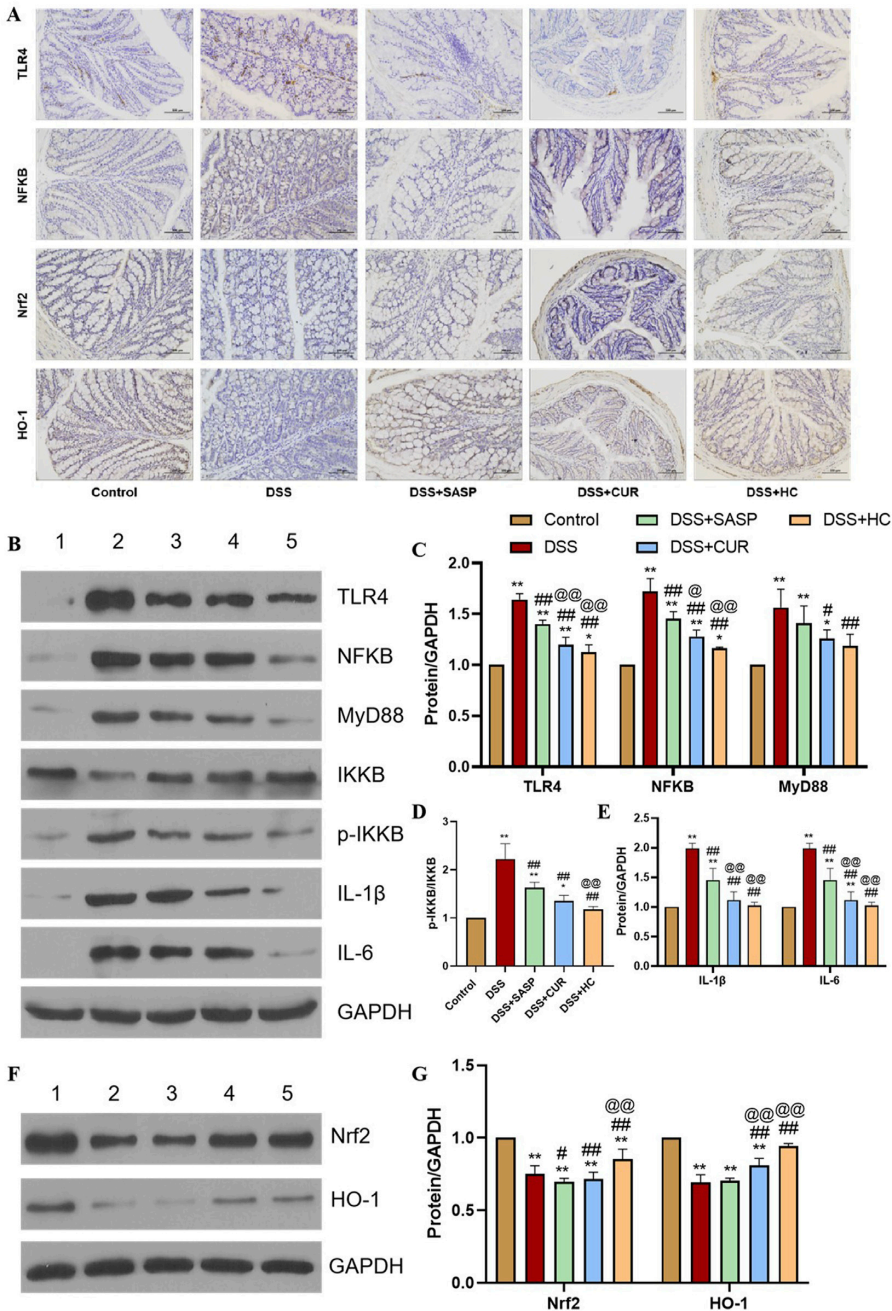
The effects of HES-CUR NPs on TLR4/NF-κB and Nrf2 antioxidant pathway-related markers. **(A)** Representative immunohistochemical staining of TLR4, NF-κB p65, Nrf2, and HO-1 expression in colonic tissues. **(B,F)** Western blot analysis of key proteins in the TLR4/NF-κB and Nrf2 antioxidant pathways (TLR4, NF-κB p65, Nrf2, HO-1; β-actin served as a loading control) (1: Control; 2: DSS; 3: DSS+SASP; 4: DSS+CUR; 5: DSS+HC). **(C–E,G)** Quantitative comparison of key proteins in the TLR4/NF-κB and Nrf2 antioxidant pathways across experimental groups.

In the TLR4/NF-κB signaling pathway, TLR4 activation triggers MyD88-dependent phosphorylation of IKKβ. This leads to IκB degradation and the subsequent nuclear translocation of NF-κB, which, in turn, drives the expression of pro-inflammatory cytokines such as IL-1β and IL-6 (Kong and Ge, 2008; Lawrence, 2009; Zhang et al., 2017). As shown in Figures 6A–E, DSS treatment significantly upregulated the expression of TLR4, NF-κB, MyD88, p-IKKβ/IKKβ, IL-1β, and IL-6 compared to the Control group (p < 0.01). In contrast, SASP, CUR, and HES-CUR NP treatments markedly downregulated these markers, with HES-CUR NPs exhibiting the most pronounced effects (p < 0.01). These observations indicated that the HES-CUR NPs attenuate colitis by inhibiting the TLR4/NF-κB pathway.

The Nrf2 antioxidant pathway plays a critical role in counteracting oxidative stress and inflammation, with HO-1 serving as its downstream effector (Li et al., 2021; Ruan et al., 2019). Here, we found that DSS treatment significantly suppressed Nrf2 and HO-1 expression relative to the Control group (p < 0.01). Conversely, SASP, CUR, and HES-CUR NP treatments robustly upregulated Nrf2 and HO-1 expression levels, with HES-CUR NPs showing the greatest efficacy (p < 0.01) (Figures 6F,G). These findings confirmed that HES-CUR NPs alleviate colitis by activating the Nrf2/HO-1 antioxidant pathway. Collectively, these results indicated that the dual modulation involving TLR4/NF-κB pathway suppression and Nrf2/HO-1 pathway activation underpins the therapeutic superiority of HES-CUR NPs in mitigating ulcerative colitis relative to the other treatments.

### 3.5 HES-CUR NPs exerted positive effects on gut beneficial bacteria flora and microbial diversity in UC model mice

**FIGURE 7:**
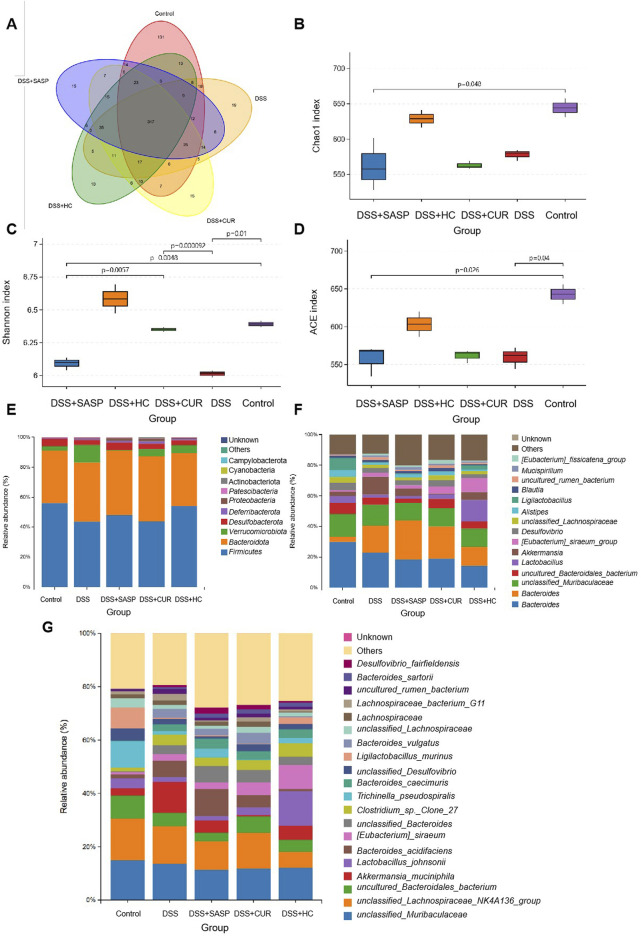
The effects of HES-CUR NPs on gut beneficial bacterias microbiota and microbial diversity in ulcerative colitis (UC) model mice. **(A)** A Venn diagram illustrating unique and shared bacterial taxa across the experimental groups. **(B–D)** Alpha diversity analysis of gut microbiota: **(B)** Chao1 index (richness), **(C)** Shannon index (diversity), and **(D)** Simpson index (evenness). **(E–G)** Relative abundance of gut microbiota at the phylum **(E)**, family **(F)**, and genus **(G)** levels.

A total of 347 shared operational taxonomic units (OTUs) were identified across all the groups. Compared to Control mice, UC (DSS-treated) model animals exhibited reduced alpha diversity index (Chao1, Shannon, ACE) values, alongside decreased abundances of beneficial bacterias such as *Ligilactobacillus murinus* and *Lactobacillus johnsonii*. In contrast, the values of these diversity indices were significantly higher in mice treated with HES-CUR NP than in UC model mice; HES-CUR NP treatment also restored the relative abundances of the two beneficial bacterias (Figure 7). These findings highlighted the ability of HES-CUR NPs to ameliorate DSS-induced gut dysbiosis and enhance microbial diversity, potentially contributing to its therapeutic efficacy in UC.

### 3.6 Histopathological analysis and inflammatory cytokine levels in UC mice under antibiotic intervention

**FIGURE 8:**
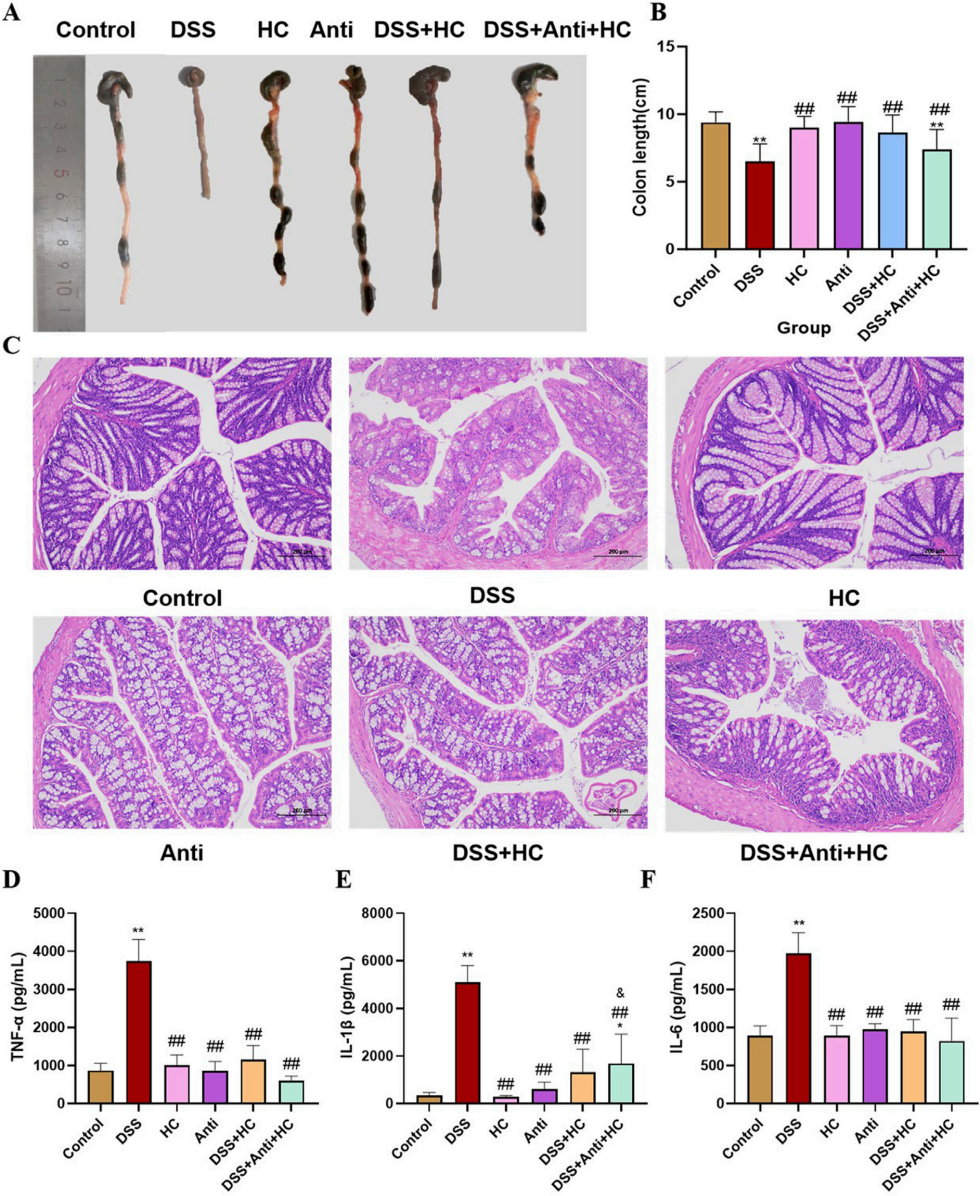
The therapeutic effects of HES-CUR NPs on ulcerative colitis (UC) model mice under antibiotic intervention. **(A,B)** Colon length alterations in mice. **(C)** Representative hematoxylin and eosin (H&E)-stained histological sections of colonic tissues (×200 magnification). **(D)** TNF-α mRNA expression. **(E)** IL-1β mRNA expression. **(F)** IL-6 mRNA expression. *p < 0.05, **p < 0.01, compared with the Control group; #p < 0.05, ##p < 0.01, compared with the DSS group; @p < 0.05, @@p < 0.01, compared with the DSS + HC group; &p < 0.05, &&p < 0.01, compared with the HC group. Similar annotations apply to subsequent figures.

DSS treatment induced significant colon shortening in mice compared to the Control group. Meanwhile, HES-CUR NP treatment partially restored colon length (p < 0.01), an effect that was significantly attenuated when accompanied by antibiotic administration (Figures 8A,B). These findings indicated that antibiotic-induced microbiota depletion compromises the therapeutic efficacy of the HES-CUR NPs. Evaluation of H&E-stained colon sections (Figure 8C) revealed the presence of severe mucosal damage, crypt distortion, and inflammatory infiltration in the DSS group. Although HES-CUR NP treatment ameliorated these pathological features, these protective effects were diminished under antibiotic intervention. Furthermore, DSS administration markedly elevated serum levels of the pro-inflammatory cytokines IL-6, IL-1β, and TNF-α (p < 0.01). While HES-CUR NP treatment significantly mitigated the DSS-induced increase in the contents of these cytokines (p < 0.05), and this anti-inflammatory effect did not diminish under antibiotic treatment (Figures 8D–F). But collectively, these results demonstrated that gut microbiota modulation is essential for the HES-CUR NP-mediated attenuation of colitis-associated inflammation and mucosal damage.

### 3.7 The impact of HES-CUR NPs on intestinal barrier function in UC model mice under antibiotic intervention

**FIGURE 9:**
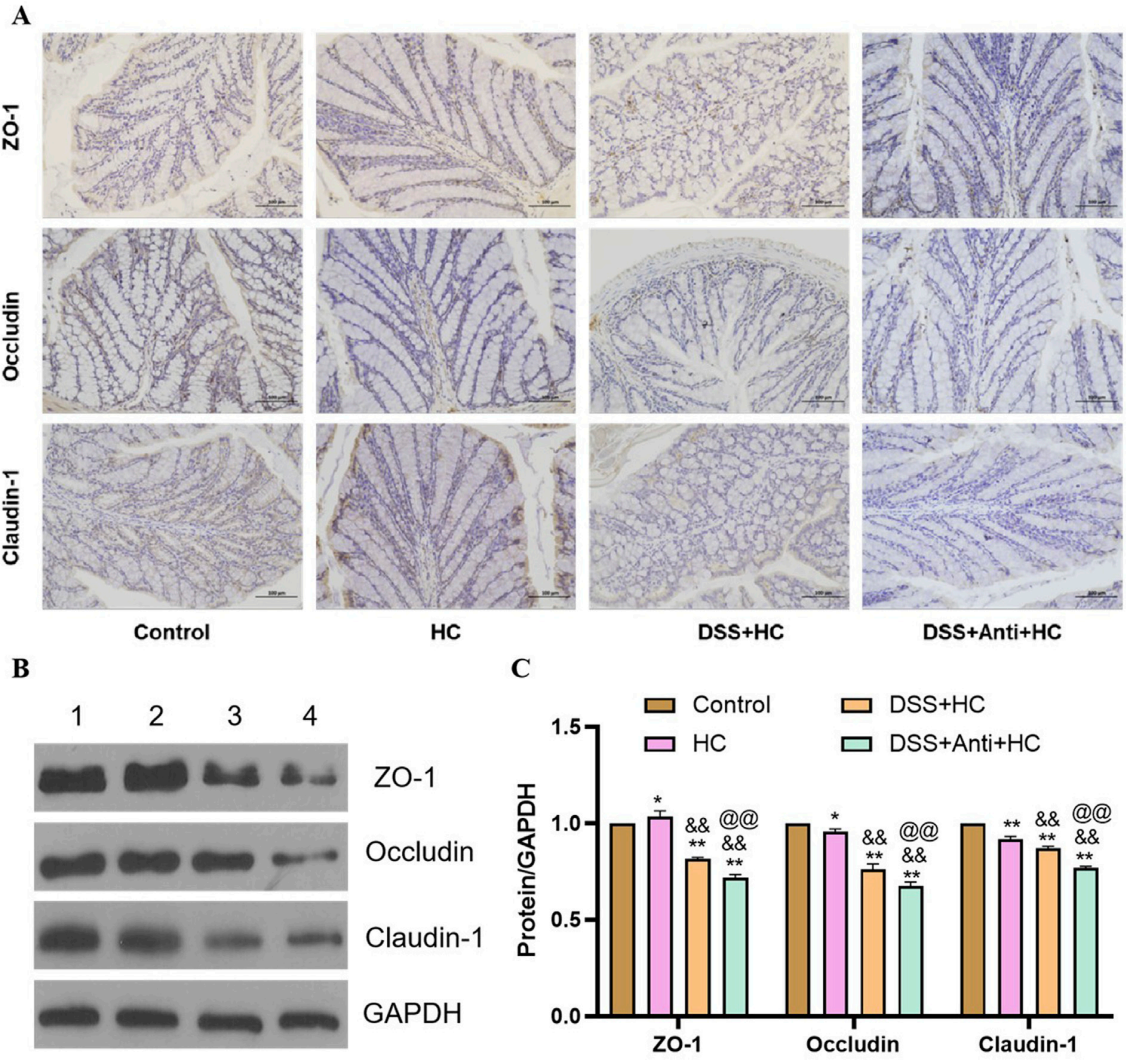
The effects of HES-CUR NPs on intestinal barrier function-related proteins in the colon of DSS-treated mice under antibiotic intervention. **(A)** Representative immunohistochemical staining of mucosal barrier proteins (ZO-1, occludin, claudin-1) in colonic tissues. **(B)** Western blot analysis of ZO-1, occludin, and claudin-1 protein expression (1: Control; 2: HC; 3: DSS+HC; 4: DSS+Anti+HC). **(C)** Quantitative comparison of ZO-1, occludin, and claudin-1 positivity across the experimental groups.

HES-CUR NP treatment markedly upregulated the levels of the tight junction proteins ZO-1, occludin, and claudin-1 in UC model animals (Figure 5). However, these restorative effects on ZO-1, occludin, and claudin-1 expression were significantly mitigated following antibiotic treatment (p < 0.05), indicating that antibiotic intervention partially abolished HES-CUR NP-mediated intestinal barrier repair (Figure 9).

### 3.8 The impact of HES-CUR NPs on the gut microbiota in UC model mice under antibiotic intervention

**FIGURE 10:**
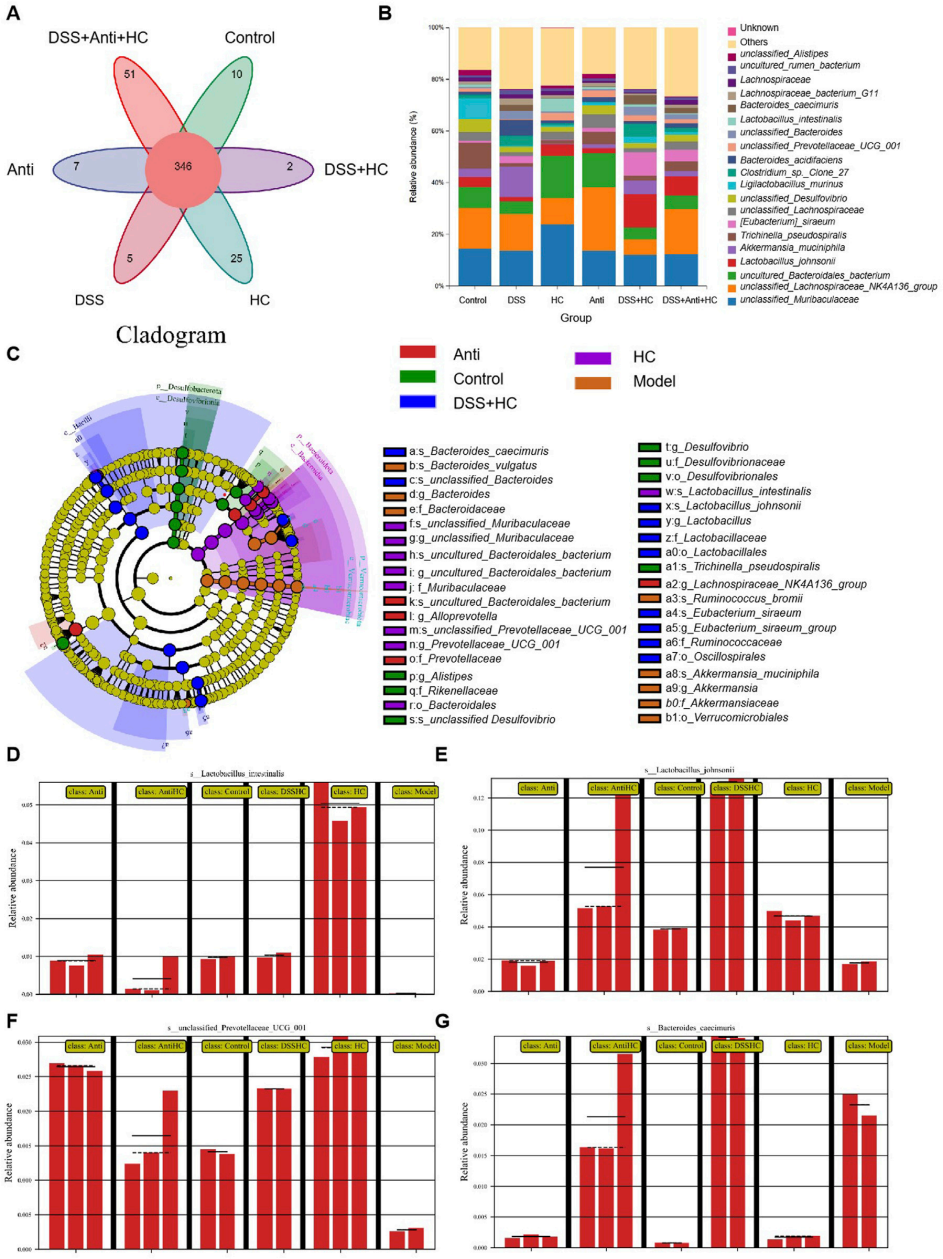
The effects of HES-CUR NPs on gut beneficial bacteria microbiota and microbial diversity in ulcerative colitis (UC) model mice under antibiotic intervention. **(A)** A Venn diagram depicting unique and shared bacterial taxa among the experimental groups. **(B)** The relative abundance of gut microbiota at the phylum level. **(C)** Phylogenetic cladogram illustrating microbial community structure. **(D–G)** Linear discriminant analysis Effect Size (LEfSe) analysis identifying differentially abundant taxa across groups (LDA score threshold >3).

As shown in Figures 10A, B, the abundances of beneficial bacterias such as *Ligilactobacillus murinus* and *Lactobacillus johnsonii* were significantly reduced in the UC model (DSS) group compared with those in the Control group. Meanwhile, HES-CUR NP treatment reversed this decline in beneficial bacteria abundance. However, antibiotic treatment attenuated the HES-CUR NP-mediated restorative effect on *Ligilactobacillus murinus* and *Lactobacillus johnsonii* abundance, indicating that the therapeutic effects of the HES-CUR NPs were at least partially dependent on the gut microbiota. Linear discriminant analysis (LDA) Effect Size (LEfSe) analysis was employed to identify differentially enriched bacterial taxa across experimental groups (Figure 10C). Distinct gut microbial structures were observed among the five groups, with purple, green, red, blue, and orange representing the HES-CUR NP, Control, DSS + HES-CUR NP, Antibiotic + HES-CUR NP, and Model groups, respectively. Circle sizes denote relative microbial abundances. Figures 10D–G further illustrate species with significant abundance differences. Beneficial taxa such as *Lactobacillus intestinalis*, *Lactobacillus johnsonii* and *unclassified_Prevotellaceae_UCG_001* were markedly reduced in the Model group compared to the Control group. HC treatment significantly restored the abundances of *L. intestinalis*, *L. johnsonii*, and *unclassified_Prevotellaceae_UCG_001* in the DSS + HC group. However, this restorative effect was diminished in the Antibiotic + HC group, underscoring the critical role of gut microbiota in mediating HC’s efficacy.

### 3.9 Correlation analysis between UC pathological indicators and gut microbiota abundance

**FIGURE 11:**
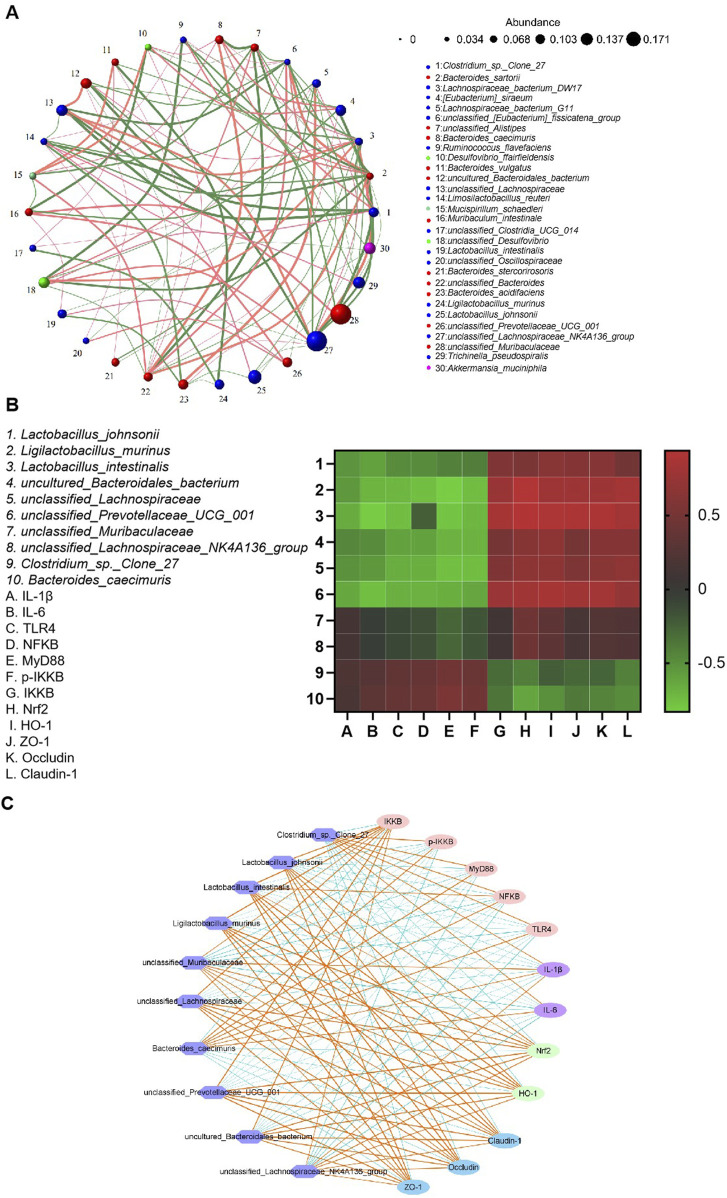
Correlation analysis between gut microbiota abundance and antioxidant/inflammatory indices. **(A)** Correlation network of gut microbial taxa. **(B)** A heatmap illustrating the correlations between microbiota abundance and biochemical indices. **(C)** Microbial co-occurrence network analysis.

Alterations in the composition of various gut bacteria were correlated with the levels of pro-inflammatory factors and signaling pathway-associated proteins. As shown in Figure 11, the abundance of *Lactobacillus johnsonii* and *Ligilactobacillus murinus* was negatively correlated with the levels of IL-1β and IL-6 but positively correlated with those of Nrf2 and HO-1. In contrast, the abundance of *Bacteroides caecimuris* showed a positive correlation with the levels of NF-κB, MyD88, and p-IKKβ, while demonstrating a negative correlation with the expression levels of the tight junction proteins claudin-1, ZO-1, and occludin. These findings underscored the critical role of the gut microbiota in the pathogenesis of UC.

## 4 Discussion

The therapeutic agents currently used for UC often induce severe side effects (Lichtenstein, 2010), stressing the need for the development of natural bioactive compounds for colitis prevention and treatment. Based on our previous work, in this study, we used a DSS-induced mouse colitis model. DSS administration resulted in colon shortening, severe ulceration, an increase in inflammatory cytokine levels, and upregulation of the mRNA levels of pro-inflammatory mediators. Notably, the oral administration of HES-CUR NPs significantly alleviated colonic inflammation, even outperforming free curcumin, likely due to the enhanced drug-loading capacity and solubility of the curcumin-loaded NPs, as demonstrated in our previous studies (Jiang et al., 2021; Chen et al., 2020). Safety assessments revealed that the nanoformulation exhibited no significant toxicity. No significant differences in spleen, liver, or thymus indices were observed between the Control and HES-CUR NP groups, indicative of the absence of pathological hypertrophy or atrophy in immune organs (spleen, thymus) or the liver. The levels of hepatic function markers (ALT, AST) remained comparable to those of the controls, confirming that there was no hepatocyte damage or inflammatory response. Plasma total protein, albumin levels, and the A/G ratio remained stable, further validating that hepatic synthesis and protein metabolism were unaltered following HES-CUR NP treatment. Analysis of H&E-stained liver sections in the HES-CUR NP treatment group showed intact hepatic lobule architecture, orderly hepatocyte arrangement, and the absence of steatosis, necrosis, or inflammatory infiltration, aligning with the results of the biochemical analysis. Combined, these findings demonstrate the efficacy and favorable safety profile of the HES-CUR NPs in a murine model of colitis.

Histopathological findings from this study revealed that DSS intake induced colonic hyperemia, edema, extensive inflammatory cell infiltration, and intestinal mucosal damage in mice, effects that may be closely associated with excessive inflammation. Neutrophils and macrophages secrete inflammatory cytokines while clearing foreign substances, such as bacteria, from the colon. The persistent overactivation and infiltration of these cytokines exacerbate tissue injury (Sklyarov et al., 2011). Notably, curcumin administration markedly attenuated colonic damage, with HES-CUR NPs demonstrating superior efficacy relative to the other treatments, likely attributable to the enhanced drug-loading capacity of the system and the increased curcumin solubility. Curcumin (CUR) exhibits inherently strong hydrophobicity, resulting in extremely low water solubility and bioavailability. By conjugating with the hydrophilic polymer hydroxyethyl starch (HES) to form an amphiphilic conjugate (HES-CUR), the water solubility of CUR is significantly enhanced. Simultaneously, as a semi-synthetic biodegradable polymer, HES protects CUR from degradation and prolongs its circulation time. The amphiphilic HES-CUR conjugate spontaneously self-assembles into nanoparticles (NPs) in aqueous solutions, a structure that increases drug-loading efficiency (DLE to 25.61%) and enables controlled release (Jiang et al., 2021). The ester bond linkage in HES-CUR endows the nanoparticles with pH-responsive properties (Yao et al., 2023), allowing rapid drug release in the acidic inflammatory microenvironment while minimizing toxicity to normal cells. These observations align with the findings of Ran et al. (Feng et al., 2023), who reported similar therapeutic effects using luteolin-enriched extracts in animal models of colitis.

Tight junction proteins in the colonic epithelium play a pivotal role in regulating intestinal mucosal permeability and maintaining the integrity of the intestinal barrier (Wang et al., 2016). The deficiency of these proteins increases intestinal permeability, elevating the risk of translocation by harmful bacteria and invasion by excessive inflammatory factors into tissues, thereby triggering the hyperactivation of intestinal immune cells and perpetuating chronic inflammation (Lechuga and Ivanov, 2017; Tan et al., 2019). Accordingly, differences in the expression and secretion of tight junction proteins serve as important biomarkers for evaluating therapeutic interventions in colitis. In this study, DSS administration significantly reduced the protein expression of ZO-1, claudin-1, and occludin in mouse colonic tissues, leading to compromised barrier function. Importantly, HES-CUR NP treatment restored tight junction protein levels and ameliorated intestinal barrier integrity, highlighting its potential as a therapeutic agent for colitis management.

The TLR4/NF-κB signaling pathway serves as a pivotal mediator of inflammatory responses in UC. Within the intestinal mucosa, TLR4 receptors recognize pathogen-associated molecular patterns (PAMPs) and damage-associated molecular patterns (DAMPs), leading to the activation of the NF-κB pathway, which, in turn, drives the production and release of pro-inflammatory cytokines, ultimately exacerbating mucosal inflammation (Zhu et al., 2022; Cao et al., 2018; Wei et al., 2023; Hu et al., 2021). Concurrently, the Nrf2/HO-1 signaling pathway, a central endogenous antioxidant defense mechanism, is closely implicated in UC pathogenesis (Loboda et al., 2016; Peng et al., 2023), representing a promising therapeutic target. In this study, mice with DSS-induced colitis exhibited significant activation of the TLR4/MyD88/NF-κB pathway alongside the suppression of Nrf2 nuclear translocation and HO-1 expression, indicative of a self-perpetuating cycle of inflammation and oxidative stress that aggravates colonic injury. Notably, HES-CUR NP intervention not only downregulated the expression of key molecules in the TLR4/NF-κB pathway but also robustly activated the Nrf2/HO-1 axis, demonstrating superior efficacy compared to free curcumin (CUR group) and the positive control drug SASP (p < 0.01). Similar mechanisms, namely, the dual modulation of the TLR4/NF-κB and Nrf2 pathways, have been reported to underlie the CeAF-mediated alleviation of inflammation (Li S. et al., 2023). The exceptional therapeutic advantage of HES-CUR NPs may stem from the targeted delivery properties of its HES-based nanocarrier. Hydrophilic HES encapsulation protects curcumin from gastric acid degradation, ensuring intact delivery to the colon, while the mucoadhesive properties of HES prolong drug retention by adhering to the colonic mucus layer; additionally, NPs preferentially accumulate at sites of inflammation, enabling the localized suppression of TLR4/NF-κB signaling and the sustained activation of the Nrf2 antioxidant pathway.

The gut microbiota make a significant contribution to intestinal health and microenvironment homeostasis, with gut dysbiosis being considered a key factor in UC pathogenesis. Studies (Llewellyn et al., 2018) have shown that gut dysbiosis contributes to UC development through multiple mechanisms, including immune cell activation, the promotion of inflammatory mediator release, and the disruption of the intestinal barrier. Patients with UC typically exhibit reduced microbial diversity, characterized by a decline in the abundance of beneficial bacteria such as *Lactobacillus* and *Bifidobacterium*, and an increase in that of harmful pathobionts. The proliferation of pathogenic bacteria exacerbates the release of inflammatory mediators, while dysbiosis impairs mucosal barrier function and increases intestinal permeability, thereby triggering or aggravating inflammation. This further disrupts the gut microbiota and microenvironment, forming a vicious cycle (Yue et al., 2019). In contrast, beneficial microbiota not only maintain intestinal homeostasis and mitigate inflammatory damage but also promote UC recovery by enhancing epithelial cell proliferation, suppressing apoptosis, and upregulating tight junction protein expression, thus preserving intestinal barrier integrity (Wu et al., 2021). In this study, gut microbial diversity and beneficial bacteria abundance were significantly reduced in UC model mice. HES-CUR NP intervention restored tight junction protein expression to between 80% and 90% of baseline levels and markedly improved microbial community structure. This barrier repair effect may be attributed to the fact that HES-CUR NPs suppress the cytotoxic effects of pro-inflammatory cytokines (e.g., TNF-α) on epithelial cells by inhibiting the NF-κB pathway and upregulating HO-1 expression, which promotes mucus layer regeneration and epithelial cell proliferation. Moreover, HES-CUR NPs increase the abundance of short-chain fatty acid (SCFA)-producing bacteria (e.g., *Lactobacillus*); SCFAs, in turn, enhance intestinal barrier function through GPR41/43 receptor activation.

## 5 Conclusion

HES-CUR NPs alleviate UC through triple mechanisms—the suppression of the TLR4/NF-κB inflammatory cascade, the activation of the Nrf2/HO-1 antioxidant pathway, and the restoration of intestinal barrier integrity, concomitant with the reshaping of gut microbiota homeostasis. Its nanoscale design significantly enhances therapeutic efficacy, offering an innovative strategy for natural compound-based UC treatment. This study not only deepens the understanding of curcumin’s multi-target mechanism of action but also opens new avenues for precision-based therapeutics centered on gut microbiota modulation.

## Data Availability

The data presented in the study are deposited in the NCBI repository, accession number PRJNA1294210; available at https://www.ncbi.nlm.nih.gov/search/all/?term=PRJNA1294210.
